# ﻿Phylogenetic analyses and morphological characters reveal two new species of *Ganoderma* from Yunnan province, China

**DOI:** 10.3897/mycokeys.84.69449

**Published:** 2021-11-12

**Authors:** Jun He, Zong-Long Luo, Song-Ming Tang, Yong-Jun Li, Shu-Hong Li, Hong-Yan Su

**Affiliations:** 1 College of Agriculture and Biological Sciences, Dali University, Dali 671003, Yunnan, China; 2 Institute of Biotechnology and Germplasm Resources, Yunnan Academy of Agricultural Sciences, Kunming 650223, China; 3 Center of Excellence in Fungal Research, Mae Fah Luang University, Chiang Rai 57100, Thailand; 4 School of science, Mae Fah Luang University, Chiang Rai 57100, Thailand

**Keywords:** Ganodermataceae, novel species, phylogeny, taxonomy

## Abstract

*Ganodermadianzhongense***sp. nov.** and *G.esculentum***sp. nov.** are proposed as two new species based on both phenotypic and genotypic evidences. *Ganodermadianzhongense* is characterized by the stipitate basidiomata, laccate and oxblood red pileus, gray white pore surface, duplex context and broadly ellipsoid basidiospores (9.0–12.5 × 6.5–9.0 μm) with coarse interwall pillars. *Ganodermaesculentum* is characterized by its basidiomata with slender stipe, white pore surface, homogeneous pileus context, and slightly truncate, narrow basidiospores (8.0–12.5 × 5.0–8.0 µm). Phylogenetic analyses were carried out based on the internal transcribed spacer (ITS), translation elongation factor 1-α (TEF1-α) and the second subunit of RNA polymerase II (RPB2) sequence data. The illustrations and descriptions for the new taxa are provided.

## ﻿Introduction

Ganodermataceae was introduced by Donk (1948) which belongs to Polyporales and the latest studies indicated that it is a monophyletic group (Costa-Rezende et al. 2020). Currently, eleven genera viz. *Amauroderma* Murril, *Amaurodermellus* Costa-Rezende, *Cristataspora* Costa-Rezende, *Foraminispora* Robled*o*, Costa-Rezende & Drechsler-Santos, *Furtadoa* Costa-Rezende, Robledo & Drechsler-Santos, *Ganoderma* P. Karst., *Haddowia* Steyaert, *Humphreya* Steyaert, *Magoderna* (Murrill) Steyaert, *Sanguinoderma* Y.F. Sun, D.H. Costa & B.K. Cui and *Tomophagus* Murrill are accepted in Ganodermataceae and supported by morphology and phylogeny (Steyaert 1972; Furtado 1981; C﻿orner 1983; Zhao and Zhang 2000; Ryvarden 2004; Thametal 2012; Costa-Rezende et al. 2017; Costa-Rezende et al. 2020; Sun et al. 2020).

*Ganoderma* P. Karst (Ganodermataceae, Polyporales) was introduced to accommodate a laccate and stipitate fungus, *Ganodermalucidum* (Curtis) P. Karst (Karsten 1881). *Ganoderma* is characterized by double-walled basidiospores with inter-wall protuberances (Karsten 1881; Moncalvo and Ryvarden 1997). There are 462 records in the Index Fungorum (http://www.Indexfungorum.org/; accessed date: 7 October 2021) and 506 records in MycoBank (http://www.mycobank.org/; accessed date: 7 October 2021). *Ganoderma* is one of the most taxonomically scrutinized genera among the Ganodermataceae and even in Polyporales (Richter et al. 2015; Costa-Rezende et al. 2020). Most *Ganoderma* species are wood decomposers, found in all temperate and tropical regions (Pilotti et al. 2004; Cao et al. 2012; Zhou et al. 2015).

*Ganoderma* has long been regarded as one of the most important medicinal fungi in the world (Paterson 2006); they have been used as medicine for over two millennia in China (Dai et al. 2009). Several *Ganoderma* species are known to be prolific sources of highly active bioactive compounds, especially polysaccharides, protein, sterols, and triterpenoids (Ahmadi and Riazipour 2007; Chan et al. 2007). These compounds are known to possess extensive therapeutic properties, such as antioxidant, antitumor, and antiviral agents, and improve sleep function (De Silva et al. 2013).

Species diversity of *Ganoderma* is abundant in China and more than 30 species have been described (Zhao and Zhang 2000; Wang et al. 2009; Cao et al. 2012; Li et al. 2015; Xing et al. 2016; Hapuarachchi et al. 2018; Liu et al. 2019; He et al. 2019; Wu et al. 2020). Yunnan province is considered as one of the hot-spots for studying biodiversity of polypores, and some new *Ganoderma* species have been described (Zhao 1989; Wang et Wu 2010; Cao and Yuan 2013).

During our investigation into the diversity of *Ganoderma* in Yunnan province, several specimens of *Ganoderma* were collected from central and southern Yunnan. Phylogenetic analysis showed that the seven collections formed two distinct lineages and can be recognized as new species, hence two new species, namely *G.dianzhongense* and *G.esculentum* are introduced based on morphology and phylogeny.

## ﻿Materials and methods

### ﻿Sample collection

Seven *Ganoderma* specimens were collected during the rainy season from July 2016 to August 2019 in Yunnan Province of China. The samples were then photographed and transported back to the laboratory where their fresh macroscopic details were described. The specimens were deposited in the herbarium of Kunming Institute of Botany Academia Sinica (KUN-HKAS).

### ﻿Morphological studies

Macro-morphological characters were described based on fresh material field notes, and the photographs provided here. Color codes are from Kornerup and Wanscher (1978). Micro-morphological data were obtained from the dried specimens and observed by using a microscope following Li et al. (2015). Sections were studied at magnification of up to 1000× using a NiKon E400 microscope and phase contrast illumination. Microscopic features and measurements were made from slide preparations stained with 5% potassium hydroxide (KOH) and 2% Melzer’s reagent. Basidiospore features, hyphal system, color, sizes and shapes were recorded and photographed. Measurements were made using the Image Frame work v.0.9.7 to represent variation in the size of basidiospores, 5% of measurements were excluded from each end of the range and extreme values are given in parentheses.

The following abbreviations are used: IKI = Melzer’s reagent, IKI– = neither amyloid nor dextrinoid, KOH = 5% potassium hydroxide, CB = Cotton Blue, CB+ = Cyanophilous (Xing et al. 2018). The abbreviation for basidiospores measurements (n/m/p) denote “n” basidiospores measured from “m” basidiomata of “p” specimens. Basidiospore dimensions (and “Q” values) are given as (a) b–*av*–c (d), where “a” represents the minimum, “d’ the biggest, “*av*” the average “b” and “c” covers a minimum of 90% of the values. “Q”, the length/width ratio of a spore in side view, and “Q_m_” for the average of all basidiospores ± standard deviation (Wang et al. 2015).

### ﻿DNA extraction, PCR amplification, and sequencing

Total genomic DNA was extracted from dried pieces of pileus with tubes with modified CTAB protocol Doyle (1987). The genes ITS, TEF1-α and RPB2 were amplified by polymerase chain reaction (PCR) technique. The primers ITS1F / ITS4, TEF1-983 / TEF1-1567, and RPB2-6f / fRPB2-7cR were used to amplify the ITS, TEF1-α, RPB2 region, respectively (White et al. 1990; Liu et al. 1999; Matheny et al. 2007). PCR reactions (25 μL) contained mixture: 2.5 μL PCR reaction buffer, 2.5 μL 0.2% BSA, 2 μL dNTP (2.5 mm), 0.5 μL each of primer, 0.2 μL 5 U/μL Taq DNA polymerase, 1–1.5 μL DNA solution and 16 μL sterilized distilled H_2_O. The PCR cycling for ITS was as follows: initial denaturation at 94 °C for 5 min, followed by 35 cycles at 94 °C for 30 sec, 53 °C for 30 sec and 72 °C for 50 sec and a final extension of 72 °C for 10 min. The PCR cycling for TEF1-α was as follows: initial denaturation at 94 °C for 5 min, followed by 35 cycles at 94 °C for 30 sec, 55 °C for 30 sec and 72 °C for 50 sec and a final extension of 72 °C for 10 min. The PCR cycling for RPB2 was as follows: initial denaturation at 94 °C for 5 min, followed by 35 cycles at 94 °C for 30 sec, 50 °C for 30 sec and 72 °C for 50 sec and a final extension of 72 °C for 10 min. The PCR products were visualized via UV light after electrophoresis on 1% agarose gels stained with ethidium bromide. Successful PCR products were sent to Sangon Biotech Limited Company (Shanghai, China), using forward PCR primers. When sequences have heterozygous INDELS or ambiguous sites, samples were sequenced bidirectionally to make contigs of the amplified regions or verify the ambiguous sites (Wang et al. 2015). Raw DNA sequences were assembled and edited in Sequencher 4.1.4 and the assembled DNA sequences were deposited in GenBank (Table 1).

### ﻿Sequencing and sequence alignment

Sequence data of three partial loci Internal transcribed spacer region (ITS), RNA polymerase II subunit 2 (RPB2), and translation elongation factor 1-alpha (TEF1-α) were used in the phylogenetic analyses. Besides the sequences generated from this study, other reference sequences were selected from GenBank for phylogenetic analyses (Table 1). Sequences were aligned using the online version of MAFFT v.7 (http://mafft.cbrc.jp/alignment/server/) (Katoh and Standley 2013) and adjusted using BioEdit v.7.0.9 by hand (Hall 1999) to allow maximum alignment and minimize gaps. Ambiguous regions were excluded from the analyses and gaps were treated as missing data. The phylogeny website tool “ALTER” (Glez-Peña et al. 2010) was used to convert the alignment fasta file to Phylip format for RAxML analysis and AliView and PAUP 4.0b 10 were used to convert the alignment fasta file to a Nexus file for Bayesian analysis (Swofford 2003). Phylogenetic analyses were obtained from Maximum Likelihood (ML) and Bayesian analysis (BI).

### ﻿Molecular phylogenetic analyses

**Figure 1.**
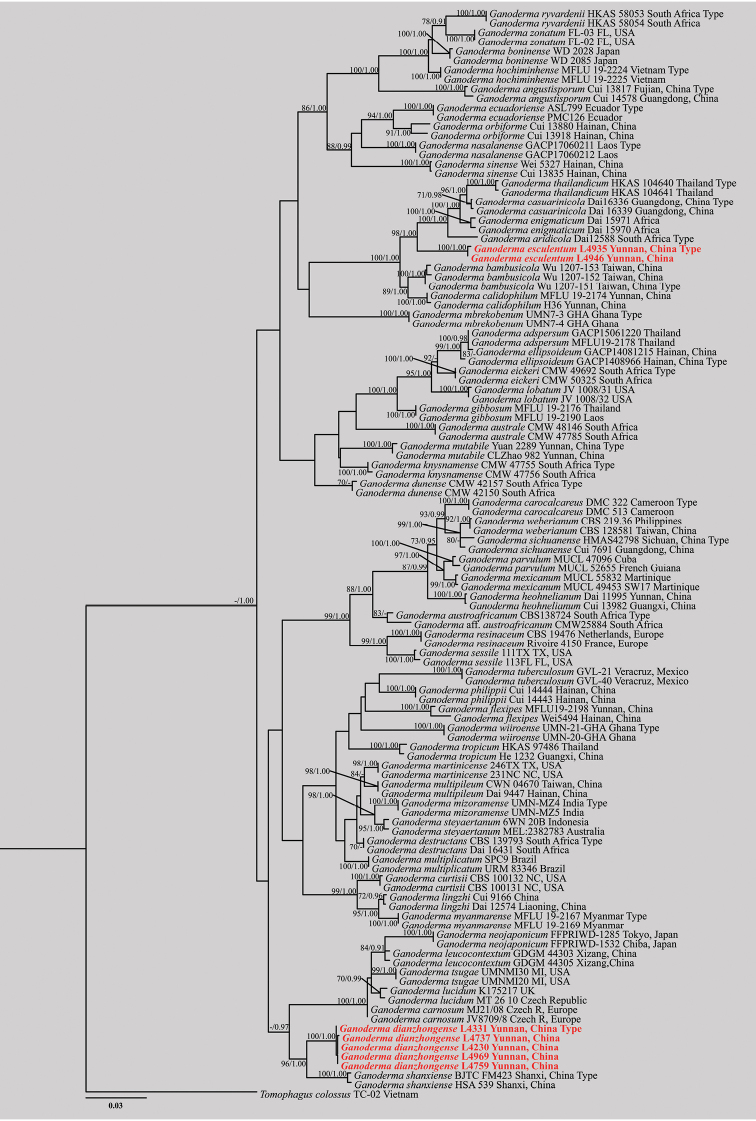
Phylogeny of the new *Ganoderma* species and related taxa based on ITS, TEF1-α and RPB2 sequence data. Branches are labeled with bootstrap values (ML) higher than 70%, and posterior probabilities (BPP) higher than 0.95. The new species are shown in bold red.

The maximum likelihood (ML) and Bayesian inference (BI) methods were used to analyze the combined dataset of ITS, TEF1-α and RPB2 sequences. Maximum likelihood analysis was conducted with RAxML-HPC2 on the CIPRES Science Gateway (Miller et al. 2010), involved 100 ML searches; all model parameters were estimated by the program. The ML bootstrap values (ML-BS) were obtained with 1000 rapid bootstrapping replicates. Maximum likelihood bootstrap values (ML) equal to or greater than 70% are given above each node (Figure 1).

**Table 1.** Species, specimens, geographic origin and GenBank accession numbers of sequences used in this study.

**Species**	**Voucher/strain**	**Origin**	**GenBank accession numbers**			**Reference**
			**ITS**	**TEF1-α**	**RPB2**	
*Ganodermaaridicola*	Dai 12588 (Type)	South Africa	KU572491	KU572502	–	Xing et al. 2016
*G.adspersum*	GACP15061220	Thailand	MK345425	MK371431	MK371437	Hapuarachchi et al. 2019
	MFLU 19-2178	Thailand	MN396653	MN423149	MN423114	Luangharn et al. 2021
*G.angustisporum*	Cui 13817(Type)	Fujian, China	MG279170	MG367563	MG367507	Xing et al. 2018
	Cui 14578	Guangdong, China	MG279171	MG367564	–	Xing et al. 2018
*G.austral*	CMW 47785	South Africa	MH571686	MH567276	–	Tchoumi et al. 2018
	CMW 48146	South Africa	MH571685	MH567283	–	Tchoumi et al. 2018
*G.austroafricanum*	CBS138724 (Type)	South Africa	KM507324	–	–	Coetzee et al. 2015
G.aff.austroafricanum	CMW25884	South Africa	MH571693	MH567296	–	Tchoumi et al. 2019
*G.bambusicola*	Wu 1207-151(Type)	Taiwan, China	MN957781	LC517941	LC517944	Wu et al. 2020
	Wu 1207-152	Taiwan, China	MN957782	LC517942	LC517945	Wu et al. 2020
	Wu 1207-153	Taiwan, China	MN957783	LC517943	LC517946	Wu et al. 2020
*G.boninense*	WD 2028	Japan	KJ143905	KJ143924	KJ143964	Zhou et al. 2015
	WD 2085	Japan	KJ143906	KJ143925	KJ143965	Zhou et al. 2015
*G.calidophilum*	MFLU 19-2174	Yunnan, China	MN398337	–	–	Luangharn et al. 2021
	H36	Yunnan, China	MW750241 ^*^	MW838997 ^*^	MW839003 ^*^	this study
*G.carnosum*	MJ 21/08	Czech R, Europe	KU572492	–	–	Xing et al. 2016
	JV 8709/8	Czech R, Europe	KU572493	–	–	Xing et al. 2016
*G.carocalcareus*	DMC 322 (Type)	Cameroon	EU089969	–	–	Douanla and Langer 2009
	DMC 513	Cameroon	EU089970	–	–	Douanla and Langer 2009
*G.casuarinicola*	Dai 16336 (Type)	Guangdong, China	MG279173	MG367565	MG367508	Xing et al. 2018
	Dai 16339	Guangdong, China	MG279176	MG367568	MG367511	Xing et al. 2018
*G.curtisii*	CBS 100131	NC, USA	JQ781848	KJ143926	KJ143966	Zhou et al. 2015
	CBS 100132	NC, USA	JQ781849	KJ143927	KJ143967	Zhou et al. 2015
*G.destructans*	CBS 139793 (Type)	South Africa	NR132919	–	–	Coetzee et al. 2015
	Dai 16431	South Africa	MG279177	MG367569	MG367512	Xing et al. 2018
*G.dunense*	CMW42157 (Type)	South Africa	MG020255	MG020227	–	Tchoumi et al. 2019
	CMW42150	South Africa	MG020249	MG020228	–	Tchoumi et al. 2019
*G.ecuadoriense*	ASL799 (Type)	Ecuador	KU128524	–	–	Crous et al. 2016
	PMC126	Ecuador	KU128525	–	–	Crous et al. 2016
*G.eickeri*	CMW 49692 (Type)	South Africa	MH571690	MH567287	–	Tchoumi et al. 2019
	CMW 50325	South Africa	MH571689	MH567290	–	Tchoumi et al. 2019
*G.ellipsoideum*	GACP1408966(Type)	Hainan, China	MH106867	–	–	Hapuarachchi et al. 2018
	GACP14081215	Hainan, China	MH106886	–	–	Hapuarachchi et al. 2018
*G.enigmaticum*	Dai 15970	Africa	KU572486	KU572496	MG367513	Xing et al. 2016
	Dai 15971	Africa	KU572487	KU572497	MG367514	Xing et al. 2016
***G.esculentum***	**L4935 (Type)**	**Yunnan, China**	MW750242 ^*^	MW838998 ^*^	MW839004 ^*^	**this study**
	**L4946**	**Yunnan, China**	MW750243 ^*^	MW838999 ^*^	–	**this study**
*G.flexipes*	Wei 5494	Hainan, China	JN383979	–	–	Cao and Yuan 2013
	MFLU 19-2198	Yunnan, China	MN398340	–	–	Luangharn et al. 2021
*G.gibbosum*	MFLU 19-2176	Thailand	MN396311	–	MN423118	Luangharn et al. 2021
	MFLU 19-2190	Laos	MN396310	–	MN423117	Luangharn et al. 2021
*G.heohnelianum*	Dai 11995	Yunnan, China	KU219988	MG367550	MG367497	Song et al. 2016
	Cui 13982	Guangxi, China	MG279178	MG367570	MG367515	Xing et al. 2018
*G.hochiminhense*	MFLU 19-2224(Type)	Vietnam	MN398324	MN423176	–	Luangharn et al. 2021
	MFLU 19-2225	Vietnam	MN396662	MN423177	–	Luangharn et al. 2021
*G.knysnamense*	CMW 47755 (Type)	South Africa	MH571681	MH567261	–	Tchoumi et al. 2019
	CMW 47756	South Africa	MH571684	MH567274	–	Tchoumi et al. 2019
*G.leucocontextum*	GDGM 44303	Xizang, China	KJ027607	–	–	Li et al. 2015
	GDGM 44305	Xizang, China	KJ027609	–	–	Li et al. 2015
*G.lingzhi*	Cui 9166	China	KJ143907	JX029974	JX029978	Cao et al. 2012
	Dai 12574	Liaoning, China	KJ143908	JX029977	JX029981	Cao et al. 2012
*G.lobatum*	JV 1008/31	USA	KF605671	MG367553	MG367499	Xing et al. 2018
	JV 1008/32	USA	KF605670	MG367554	MG367500	Xing et al. 2018
*G.lucidum*	K 175217	UK	KJ143911	KJ143929	KJ143971	Zhou et al. 2015
	MT 26/10	Czech Republic	KJ143912	KJ143930	–	Zhou et al. 2015
*G.martinicense*	231NC	NC, USA	MG654182	MG754736	–	Loyd et al.2018
	246TX	TX, USA	MG654185	MG754737	MG754858	Loyd et al.2018
*G.mbrekobenum*	UMN7-3 GHA (Type)	Ghana	KX000896	–	–	Crous et al. 2016
	UMN7-4 GHA	Ghana	KX000898	–	–	Crous et al. 2016
*G.mexicanum*	MUCL 49453 SW17	Martinique	MK531811	MK531825	MK531836	Cabarroi-Hernández et al. 2019
	MUCL 55832	Martinique	MK531815	MK531829	MK531839	Cabarroi-Hernández et al. 2019
*G.mizoramens*e	UMN-MZ4 (Type)	India	KY643750	–	–	Crous et al. 2017
	UMN-MZ5	India	KY643751	–	–	Crous et al. 2017
*G.multipileum*	CWN 04670	Taiwan, China	KJ143913	KJ143931	KJ143972	Zhou et al. 2015
	Dai 9447	Hainan, China	KJ143914	–	KJ143973	Zhou et al. 2015
*G.multiplicatum*	SPC9	Brazil	KU569553	–	–	Bolaños et al. 2016
	URM 83346	Brazil	JX310823	–	–	Bolaños et al. 2016
*G.mutabile*	CLZhao 982	Yunnan, China	MG231527	–	–	GenBank
	Yuan 2289(Type)	Yunnan, China	JN383977	–	–	Cao and Yuan 2013
*G.myanmarense*	MFLU 19-2167 (Type)	Myanmar	MN396329	–	–	Luangharn et al. 2021
	MFLU 19-2169	Myanmar	MN396330	–	–	Luangharn et al. 2021
*G.nasalanense*	GACP17060211 (Type)	Laos	MK345441	–	–	Hapuarachchi et al. 2019
	GACP17060212	Laos	MK345442	–	–	Hapuarachchi et al. 2019
*G.neojaponicum*	FFPRI WD-1285	Tokyo, Japan	MN957784	–	–	Wu et al. 2020
	FFPRI WD-1532	Chiba, Japan	MN957785	–	–	Wu et al. 2020
*G.orbiforme*	Cui 13918	Hainan, China	MG279186	MG367576	MG367522	Xing et al. 2018
	Cui 13880	Hainan, China	MG279187	MG367577	MG367523	Xing et al. 2018
*G.parvulum*	MUCL 47096	Cuba	MK554783	MK554721	MK554742	Cabarroi-Hernández et al. 2019
	MUCL 52655	French Guiana	MK554770	MK554717	MK554755	Cabarroi-Hernández et al. 2019
*G.philippii*	Cui 14443	Hainan, China	MG279188	MG367578	MG367524	Xing et al. 2018
	Cui 14444	Hainan, China	MG279189	MG367579	MG367525	Xing et al. 2018
*G.resinaceum*	Rivoire 4150	France, Europe	KJ143915	–	–	Zhou et al. 2015
	CBS 19476	Netherlands, Europe	KJ143916	KJ143934	–	Zhou et al. 2015
*G.ryvardenii*	HKAS 58053 (Type)	South Africa	HM138670	–	–	Kinge et al. 2011
	HKAS 58054	South Africa	HM138671	–	–	Kinge et al. 2011
*G.sessile*	111TX	TX, USA	MG654306	MG754747	MG754866	Loyd et al.2018
	113FL	FL, USA	MG654307	MG754748	MG754867	Loyd et al.2018
*G.shanxiense*	BJTC FM423(Type)	Shanxi, China	MK764268	MK783937	MK783940	Liu et al. 2019
	HSA 539	Shanxi, China	MK764269	–	MK789681	Liu et al. 2019
*G.sichuanense*	HMAS42798 (Type)	Sichuan, China	JQ781877	–	–	Cao et al. 2012
	Cui 7691	Guangdong, China	JQ781878	–	–	Cao et al. 2012
*G.sinense*	Wei 5327	Hainan, China	KF494998	KF494976	MG367529	Xing et al. 2018
	Cui 13835	Hainan, China	MG279193	MG367583	MG367530	Xing et al. 2018
*G.steyaertanum*	MEL:2382783	Australia	KP012964	–	–	GenBank
	6 WN 20B	Indonesia	KJ654462	–	–	Glen et al. 2014
*G.thailandicum*	HKAS 104640 (Type)	Thailand	MK848681	MK875829	MK875831	Luangharn et al. 2019
	HKAS 104641	Thailand	MK848682	MK875830	MK875832	Luangharn et al. 2019
*G.tropicum*	He 1232	Guangxi, China	KF495000	KF494975	MG367531	Xing et al. 2016
	HKAS 97486	Thailand	MH823539	–	MH883621	Luangharn et al. 2021
*G.tsugae*	UMNMI20	MI, USA	MG654324	MG754764	–	Loyd et al.2018
	UMNMI30	MI, USA	MG654326	MH025362	MG754871	Loyd et al.2018
*G.tuberculosum*	GVL-21	Veracruz, Mexico	MT232639	–	–	Espinosa-García et al. 2021
	GVL-40	Veracruz, Mexico	MT232634	–	–	Espinosa-García et al. 2021
*G.weberianum*	CBS 128581	Taiwan, China	MK603805	MK636693	MK611971	Cabarroi-Hernández et al. 2019
	CBS 219.36	Philippines	MK603804	MK611974	MK611972	Cabarroi-Hernández et al. 2019
*G.wiiroense*	UMN-21-GHA (Type)	Ghana	KT952363	–	–	Crous et al. 2015
	UMN-20-GHA	Ghana	KT952361	–	–	Crous et al. 2015
***G.dianzhongense***	**L4331(Type)**	**Yunnan, China**	MW750237 ^*^	MW838993 ^*^	MZ467043 ^*^	**this study**
	**L4230**	**Yunnan, China**	MW750236 ^*^	MW838992 ^*^	–	**this study**
	**L4737**	**Yunnan, China**	MW750238 ^*^	MW838994 ^*^	MW839000 ^*^	**this study**
	**L4759**	**Yunnan, China**	MW750239 ^*^	MW838995 ^*^	MW839001 ^*^	**this study**
	**L4969**	**Yunnan, China**	MW750240 ^*^	MW838996 ^*^	MZ467044 ^*^	**this study**
*G.zonatum*	FL-02	FL, USA	KJ143921	KJ143941	KJ143979	Zhou et al. 2015
	FL-03	FL, USA	KJ143922	KJ143942	KJ143980	Zhou et al. 2015
*Tomophaguscolossus*	TC-02	Vietnam	KJ143923	KJ143943	–	Zhou et al. 2015

*Newly generated sequences for this study. Bold font = new species.

Bayesian analysis was performed with MrBayes v3.2 (Ronquist et al. 2012), with the best-fit model of sequence evolution estimated with MrModeltest 2.3 (Nylander et al. 2008) to evaluate posterior probabilities (PP) (Rannala and Yang 1996; Zhaxybayeva and Gogarten 2002) by Markov Chain Monte Carlo (MCMC) sampling. Six simultaneous Markov chains were run for 10,000,000 generations, trees were sampled every 500^th^ generation, and 2,000 trees were obtained. The first 5000 trees, representing the burn-in phase of the analyses, were discarded, while the remaining 1500 trees were used for calculating posterior probabilities in the majority rule consensus tree (the critical value for the topological convergence diagnostic is 0.01).

The phylogenetic tree was visualized with FigTree version 1.4.0 (Rambaut 2012) and made in Adobe Illustrator CS5 (Adobe Systems Inc., USA). Sequences derived in this study were deposited in GenBank (http://www.ncbi.nlm.nih.gov). The final sequence alignments and the phylogenetic trees are available at TreeBase (http://www.treebase.org, accession number: 28875).

## ﻿Results

### ﻿Phylogenetic analyses

The dataset composed of ITS, TEF1-α and RPB2 genes, comprising a total of 2092 characters including gaps, ITS (1–656 bp), TEF1-α (657–1192 bp) and RPB2 (1193–2092 bp), including 57 taxa with *Tomophaguscolossus* (Fr.) C.F. Baker as the outgroup taxon (Wang et al. 2009; Cao et al. 2012). Best model for the combined 3-gene dataset estimated and applied in the Bayesian analysis was GTR+I+G, lset nst = 6, rates = invgamma; prset statefreqpr = dirichlet (1,1,1,1). The phylogenetic analysis of ML and BI produce similar topology. The combined dataset analysis of RAxML generates a best-scoring tree (Figure 1), with the final ML optimization likelihood value of -13861.891117. The aligned matrix had 993 distinct alignment patterns, with 38.83% completely undetermined characters or gaps. The base frequency and rate are as follows: A = 0.215319, C = 0.266028, G = 0.260220, T = 0.258433; rate AC = 0.885915, AG = 5.586021, AT = 0.936363, CG = 1.205084, CT = 6.595971, GT = 1.000000; gamma distribution shape: α = 0.246210. Bootstrap support values with a maximum likelihood (ML) greater than 70%, and Bayesian posterior probabilities (BPP) greater than 0.95 are given above the nodes (Figure 1).

Phylogenetic analysis showed that five collections clustered together with high bootstrap support, forming a clade sister to *G.shanxiense* with strong bootstrap support (ML-BS = 96%, BPP = 1.00, Figure 1). Two other collections clustered with *G.aridicola*, *G.bambusicola*, *G.casuarinicola*, *G.calidohilum*, *G.enigmaticum* and *G.thailandicum* (ML-BS = 100%, BPP = 1.00), but forming as a distinct lineage.

### ﻿Taxonomy

#### ﻿ Ganoderma dianzhongense

Taxon classificationFungiPolyporalesPolyporaceae

J. He, H.Y. Su & S.H. Li sp. nov.

7822BDB2-515A-56F5-BCDD-BF8EC241821D

Index Fungorum number: 558822

841408

Figure 2

##### Diagnosis.

*Ganodermadianzhongense* is characterized by its mesopodal basidiomata, oxblood red to violet brown pileus surface, melon seed kernel-shaped and broadly ellipsoid basidiospores.

##### Holotype.

**China.** Yunnan Province, Kunming City, Luquan County, on the rotten broad-leaved trees, alt. 2480 m, Shu-Hong Li, 8 Sept. 2016, L4331 (HKAS 110005).

##### Etymology.

The epithet ‘dianzhong’ refers to central Yunnan province in Chinese, where the holotype was collected.

##### Description.

**Basidiomata** annual, stipitate, sub-mesopodal to mesopodal or with the back sides fused, coriaceous to woody. **Pileus** single, suborbicular to reniform, up to 4.8–13.1 cm diam., 1.1 cm thick, weakly to strongly laccate, glossy and shiny, oxblood red (9E7) to violet brown (11F8), smooth, and covered by a thin hard crust, concentrically zonate or azonate. **Margin** distinct, slightly obtuse. **Stipe** 9.0–17.7 × 1.1–1.9 cm, central, cylindrical, strongly laccate, dark red brown (11C8) to purplish (14A8) or almost blackish red-brown (10F4), fibrous to woody. **Context** up to 0.4 cm thick, duplex; lower layer dark brown (8F8), fibrous, composed of coarse loose fibrils; upper layer putty (4B2); corky to woody, bearing distinct concentric growth zones, without black melanoid band. **Tubes** woody hard, grayish brown, up to 0.9 cm long, unstratified. **Pore** 4–6 per mm, round to angular, dissepiments slightly thick, entire; pore surface grey white to lead gray (2D2), turning light buff when dust (5D1).

**Hyphal system trimitic.** Generative hyphae 2.0–3.5 μm in diameter, colorless, thin-walled, clamp connections present; skeletal hyphae 3.0–6.0 μm in diameter, subthick-walled to solid, non-septate, arboriform with few branches, yellowish to golden-yellow; binding hyphae 1–2.5 μm in diameter, thick-walled, frequently branched, interwoven, hyaline to yellowish, scarce; all the hyphae IKI–, CB+; tissues darkening in KOH.

**Pileipellis** a crustohymeniderm, cells 20–45 × 5.5–7.5 μm, clavate to cylindrical, entire or rarely with one lateral protuberance, thick-walled, without granulations in the apex, golden-yellow to yellowish-brown, thick-walled, moderately amyloid at maturity.

**Basidiospores** (80/6/3) (9.0) 10–***11*.*0***–12.0 (12.5) × (6.5) 7.0–***7*.*9***–8.5 (9.0) μm, Q = (1.12) 1.25–1.55 (1.63), Q_m_ = 1.40±0.09 (including myxosporium); holotype: (40/2/1) 10.0–***10*.*9***–12 × 7.0–***7*.*9***–8.5 (9.0) μm,Q = (1.20) 1.25–1.52, Q_m_ = 1.39±0.08 (including myxosporium). mostly melon seed-shaped at maturity to broadly ellipsoid, usually with one end tapering and obtuse at maturity, with apical germ pore, yellowish to medium brown, IKI–, CB+, inamyloid; perisporium wrinkled, double-walled, with coarse interwall pillars. **Basidia** widely clavate to utriform, hyaline, with a clamp connection and four sterigmata, 11–19 ×10–13µm; basidioles pear-shaped to fusiform, 10–15 × 8–12 µm.

**Figure 2.**
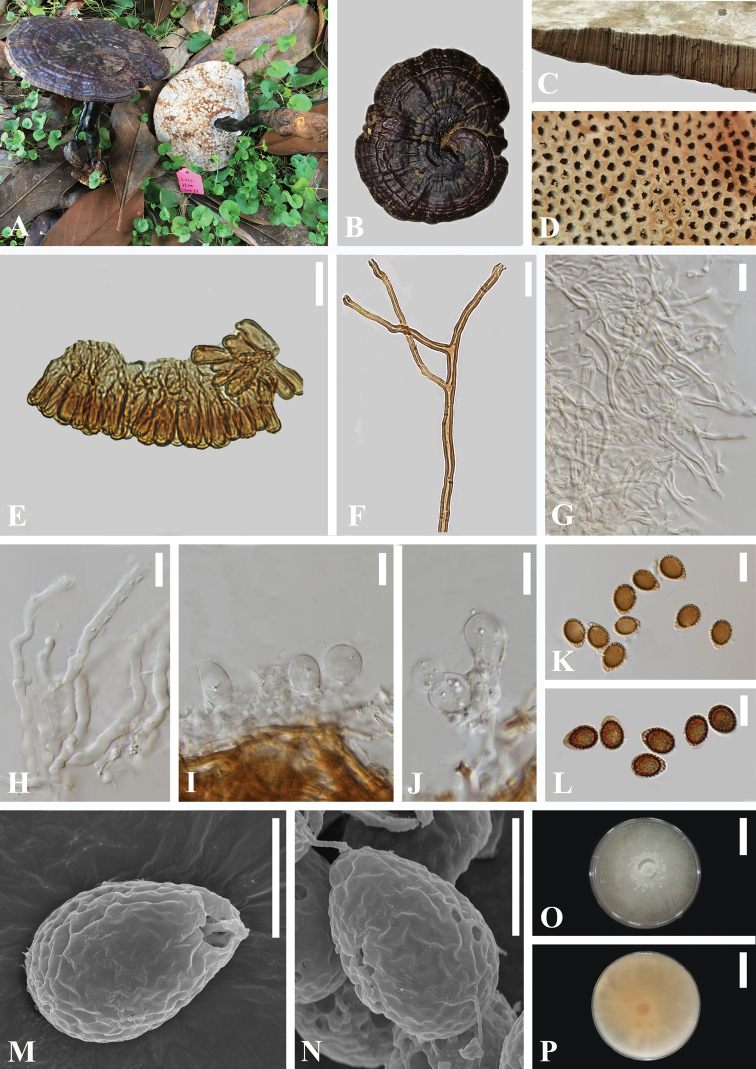
*Ganodermadianzhongense* (HKAS 110005, holotype) **A** basidiomata **B** upper surface **C** cut side of pileus **D** pore surface **E** sections of pileipellis (LM) **F** skeletal hyphae from context (LM) **G** binging hyphae from tubes (LM) **H** generative hyphae from tubes (LM) **I-J** basidia and basidioles (LM) **K-L** basidiospores (LM) **M-N** basidiospores (SEM) **O-P** culture after incubation at 28 °C for 8 days. Scale bars: 20 mm (**O, P**); 10 µm (**E-L**); 5 µm (**M, N**). Photographs Jun He.

##### Habit.

Scattered, during fall, decaying wood of broad-leaved trees including *Quercus* sp. Currently, only known from central Yunnan province, China.

##### Additional specimens examined.

**China.** Yunnan province, Shilin County, alt. 2109m, Jun He, 28 Aug., 2019, L4969 (HKAS 112719); Songming County, alt. 2204m, Shu-Hong Li, 8 Jul., 2016, L4230 (HKAS 112716); Wuding County, alt. 2295m, Shu-Hong Li, 24 Jul., 2019, L4737 (HKAS 112717); ibid, alt. 2432m, Jun He, 26 Jul., 2019, L4759 (HKAS 112718).

#### ﻿ Ganoderma esculentum

Taxon classificationFungiPolyporalesPolyporaceae

J. He & S.H. Li sp. nov.

30AAEA53-95E1-5A9A-8A3E-B99120337299

Index Fungorum number: 558823

841409

Figure 3

##### Diagnosis.

*Ganodermaesculentum* is characterized by its strongly laccate chocolate brown pileus surface, slender stipe and narrow ellipsoid basidiospores.

##### Holotype.

**China**. Yunnan Province, Honghe City, Mengzi County, on a decaying wood log, alt. 1370 m, Jun He, 26 Aug., 2019, L4935 (HKAS 110006).

##### Etymology.

The epithet ‘esculentum’ refers to this species named after a food.

##### Description.

**Basidiomata** annual, stipitate, pleuropodal, laccate, woody-corky. **Pileus** single, sub-orbicular to reniform to spathulate, up to 2.8–8.0 × 2.0–4.5 cm diam, 0.75 cm thick at the base, slightly convex to applanate; surface glabrous, rugose to radially rugose, strongly laccate, not cracking, with a hard crust, difficult to penetrate with the fingernail; surface brownish-black (6C8) to chocolate brown (6F4), almost homogeneous in the adult. **Margin** grayish orange(6B5) to concolorous, entire, acute to obtuse, smooth to sulcate. **Stipe** 10.0–17.5 × 0.5–1.0 cm, dorsally lateral to nearly dorsal, sub-cylindrical, solid, surface smooth, very shiny, dark brown (8F8) almost black, darker than pileus, fibrous to woody. **Context** up to 0.2 cm thick, composed of coarse loose fibrils, dark brown (8F8), with black melanoid band. **Tubes** 0.2–0.5 cm long, dark brown, woody hard, unstratified. **Pore** 5–8 per mm, circular or sub-circular, woody; pore surface white when fresh, darkening to soot brown(5F5) when aging and drying.

**Hyphal system trimitic.** Generative hyphae 1.5–3.0 μm in diameter, colorless, thin-walled, clamp connections present; skeletal hyphae 3.5–5.5 μm in diameter, thick-walled to solid, non-septate, arboriform or not, non-branched or with a few branches in the distal end, golden brown; binding hyphae 1.0–3.0 μm in diameter, thick-walled, much-branched, arboriform, hyaline to yellowish, scarce; all the hyphae IKI–, CB+; tissues darkening in KOH.

**Pileipellis** a crustohymeniderm, cells 20–55 × 10–15 μm, narrowly clavate to tubular, generally smooth, slightly thick-walled to thick-walled with a wide lumen, occasionally expanded at the apex, without granulations, entire, yellowish to leather brown, weakly to strongly amyloid.

**Basidiospores** (40/3/2) (8.0) 9.0–***10*.*6***–12.5 × (5.0) 5.5–***6*.*6***–7.5 (8.0) μm, Q = (1.15) 1.34–***1*.*62***–2.01 (2.06), Q_m_ = 1.62±0.19 (including myxosporium); holotype: (20/2/1) 9.0–***10*.*6***–12.5 × (5.0) 5.5–***6*.*5***–7.0 (8.0) μm, Q = (1.34) 1.45–***1*.*64***–1.83 (2.06), Q_m_ = 1.64±0.15 (including myxosporium). narrow ellipsoid to truncate, slightly visible apical germ pore, brownish orange to light brown, IKI–, CB+, inamyloid; with a brown eusporium bearing fine, overlaid by a hyaline myxosporium, with interwall pillars. **Basidia** not observed.

**Figure 3.**
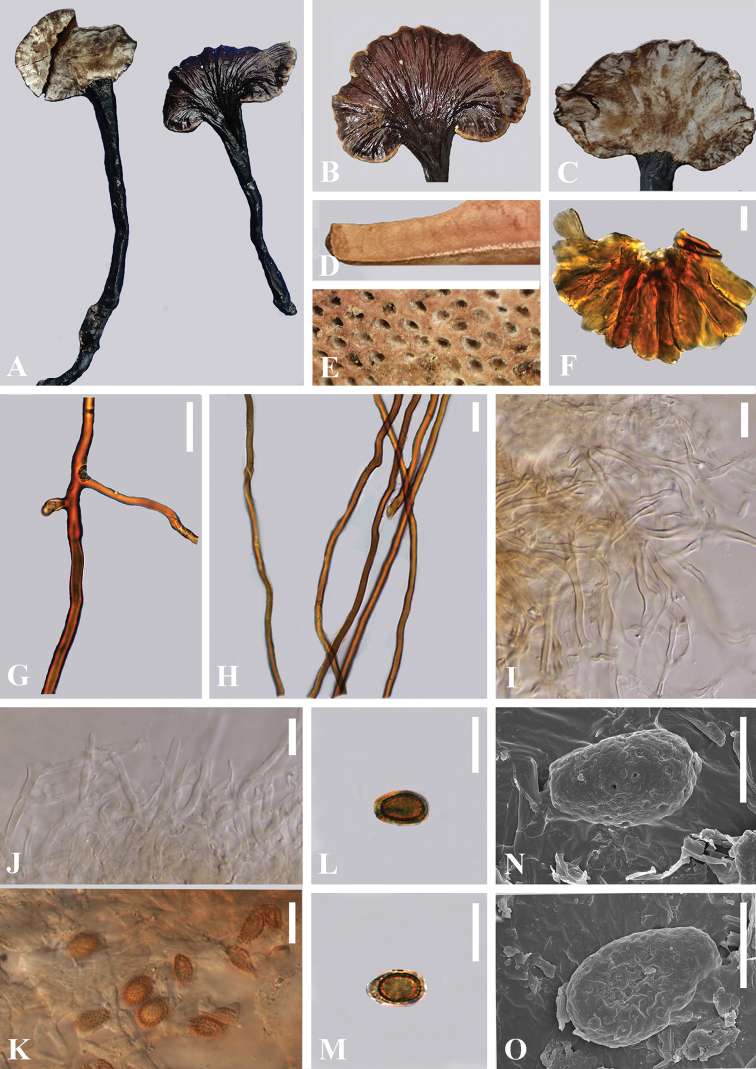
*Ganodermaesculentum* holotype (HKAS 110006) **A** basidiomata **B** upper surface **C** lower surface **D** cut side of pileus **E** pore surface **F** sections of pileipellis (LM) **G, H** skeletal hyphae from context (LM) **I** binging hyphae from tubes (LM) **J** generative hyphae from tubes (LM) **K–M** basidiospores (LM) **N, O** basidiospores (SEM). Scale bars: 20 µm (**H**); 10 µm (**F, G, I-M**); 5 µm (**N, O**). Photographs Jun He.

##### Habit.

On decaying hardwood trees or bamboo roots, accompanied in humus rich soil with over heavily rotted litter on the ground.

##### Additional specimens examined.

**China.** Yunnan province, Mengzi City, Xinansuo Town, alt. 1328m, Jun He, 26 Aug., 2019, L4946 (HKAS 112720).

## ﻿Discussion

Ganodermataceae is a large family of polypores, and has received great attention from mycologists for over many decades. However, species identification and circumscriptions have been unclear and taxonomic segregation of the genera has been controversial because of different viewpoints among mycologists (Moncalvo et al. 1995; Moncalvo and Ryvarden 1997; Costa-Rezende et al. 2020). Ganodermataceae was treated as a synonym of Polyporaceae and classify the genus *Ganoderma* into Polyporaceae by Justo et al. (2017). Later, Cui et al. (2019) excluded *Ganoderma* from Polyporaceae, due to *Ganoderma* having unique double-walled basidiospores. In addition, recent studies have clarified someuncer-tainties of generic delimitation and classification of polypores with ganodermatoid basidiospores, and proved that Ganodermataceae is a monophyletic group (Costa-Rezende et al. 2020). More collections of this family are needed in order to estimate the attributes of this taxon better.

In the phylogenetic inferences, *Ganodermadianzhongense* is sister to *G.shanxiense*, which is known from the northern Shanxi province in China (Figure 1). Morphologically, both species share similar characters of the mesopodal basidiomata, suborbicular to reniform pileus, and broadly ellipsoid basidiospores (Table 2). However, *G.shanxiense* differs from *G.dianzhongense* in having a red to reddish-brown pileus surface, wider basidiospores (11.0–13.0 × 8.0–9.5 μm), and narrower skeletal hyphae (2.5–5.0 μm, Liu et al. 2019).

*Ganodermadianzhongense* resembles *G.sinense* and *G.orbiforme* in having suborbicular pileus (Table 2). However, *G.sinense* is characterized by wider basidiospores (9.5–13.4 × 7.0–10.2 μm) and slightly longitudinally crested basidiospores (Wang and Wu 2007) and a uniformly brown to dark brown context. *Ganodermaorbiforme* has a purplish black to light brown pileus, a variably brown context, irregularly digitated pileipellis cells, and ellipsoid to ovoid basidiospores (6.9–10.6 × 3.6–5.7 μm) with fine and short echinulae, and a subtropical to tropical distribution (Wang et al. 2014). *Ganodermaorbiforme* is also phylogenetically unrelated (Figure 1).

In our multi-locus phylogeny analysis (Figure 1), *G.aridicola*, *G.bambusicola*, *G.casuarinicola*, *G.calidohilum*, *G.enigmaticum*, *G.mbrekobenum*, *G.thailandicum* and *G.esculentum* formed a distinct lineage, and was clearly separated from other *Ganoderma* species. It is easy to distinguish them from the morphological characteristics. *Ganodermabambusicola* has a longer pileipellis (35–65 × 8–16 μm) and wider basidiospores than those of *G.esculentum* (10.0–13.0 × 6.5–8.0 µm, Wu et al. 2020). *Ganodermaaridicola* can be easily distinguished from *G.esculentum* by the sessile basidiomata and a fuscous to black pileus surface (Xing et al. 2016). *Ganodermacasuarinicola* differs from *G.esculentum* by the latter has smaller basidiospores (8.3–11.5 × 4.5–7.0 µm, Xing et al. 2018), grayish brown longer pores and sectorial to shell-shaped pileus. *Ganodermaenigmaticum* mainly differs from *G.esculentum* by its golden yellow pileus surface, narrower basidiospores (8.0–11.0 × 3.5–6.0 µm, Coetzee et al. 2015) and causes root and butt rot of living and dead trees. *Ganodermathailandicum* can be distinguished from *G.esculentum*, by its brownish-red pileus surface without radially rugose, narrowly clavate pileipellis cells with tuberculate and smaller basidiospores (6.8–10.2 × 5.8–7.7 µm, Luangharn et al. 2019). *Ganodermambrekobenum* can be differentiated from *G.esculentum* by its woody to corky texture when dried, with ovoid basidiospores (25.0–57.0 × 6.0–12.0µm, Crous et al. 2016). *Ganodermacalidophilum* has a larger diameter binding hypha (2.4–5.2 µm) than *G.esculentum* (1.0–3.0 μm) and *G.calidophilum* has larger basidiospores (7.3–14.6 × 5.3–9.6 µm, Zhao et al. 1979; Luangharn et al. 2021) than *G.esculentum* (including myxosporium).

**Table 2.** Morphological comparison of *Ganodermadianzhongense* sp. nov., and *G.esculentum* sp. nov., with their closest relatives in the combined phylogeny.

Species	Shape	Context	Pileipellis cells	Pores	Basidiospores (μm)	Reference
*Ganodermaaridicola*	sessile dimidiate	context corky, fuscous, black melanoid band absent	moderately amyloid at maturity, 30–55 × 5–8 μm	6–8 per mm	9.7–11.2 × 7.0–7.8	Xing et al. 2016
*G.bambusicola*	stipitate, reniform to semicircular	context fairly homogeneous, brownish,1–2 mm thick	clavate or cylindrical, 35–65 × 8–16 μm	5–6 per mm	11.0–12.5 × 6.5––7.5	Wu et al. 2020
*G.carnosum*	laterally to rarely eccentrically stipitate, dimidiate, orbicular to reniform	whitish and soft-corky context	amyloid elements up to 75 μm from clamp to the ape	3–4 per mm	10.0–13 × 7.0–8.5	Patouillard 1889
*G.calidophilum*	stipitate, round or half-round	duplex context, 0.1–0.3 cm thick	–	4–6 per mm	10.0–13.0 × 6.2–8.7	Zhao et al. 1979
*G.casuarinicola*	stipitate, sectorial to shell-shaped	context corky, black melanoid band absent.	moderately amyloid at maturity, 40–70 × 5–13μm	4–6 per mm	8.3–11.5 × 4.5–7.0	Xing et al. 2018
*G.dianzhongense*	stipitate, suborbicular to reniform	dark brown context, black melanoid band present	amyloid elements, 20–45 × 5.5–7.5 μm	5–8 per mm	9.0–12.5 × 6.5–9.0	this study
*G.enigmaticum*	stipitate globular pileus	context soft, dark brown	amyloid elements 20–46 × 5.5–9 um	3–5 per mm	8.0–11.0 × 3.5–6.0	Coetzee et al. 2015
*G.esculentum*	stipitate, reniform to spathulate	dark brown context, without black melanoid bands	weakly to strongly amyloid, 20–55 × 10–15 μm	4–6 per mm	8.0–12.5 × 5.0–8.0	this study
*G.kunmingense*	stipitate, spathulate or half-round	context wood color	–	4 per mm	7.5–10.5 × 6.0–9.0	Zhao 1989
*G.lucidum*	stipitate to sessile	thinner context of white to slightly cream color context	amyloid hyphal end cells up to 7–11 μm diam	4–5 per mm	7.7–11.5 × 5.2–8.4	Ryvarden and Gilbertson 1993
*G.leucocontextum*	stipitate, reniform to flabelliform	thinner context of white to slightly cream color	amyloid elements 30–60 × 8–10 μm	4–6 per mm	9.5–12.5 × 7.0–9.0	Li et al. 2015
*G.mbrekobenum*	stipitate, maroon to liver brown	–	–	4–6 per mm	8.0–11.5 × 6.0–8.0	Crous et al. 2016
*G.neojaponicum*	stipitate, reniform to suborbicular	0.5 cm thick, duplex	brownish orange, clavate like cells	3–5 per mm	9.1–13.5 × 5.7–8.9	Imazeki et al. 1939
*G.orbiforme*	sessile, flabelliform or spathulate	context up to 0.4–1.0 cm thick, triplex	composed of apically acanthus like branched cells	4–6 per mm	7.1–11.8 × 5.2–7.7	Ryvarden 2000
*G.sinense*	stipitate, dimidiate, suborbicular	soft and fibrous, dark brown	clavate like cells, dextrinoid	5–6 per mm	9.5–13.8 × 6.9–8.7	Zhao et al. 1979
*G.shanxiense*	stipitate, reniform to dimidiate	brown context	25–30 × 7.5–8.5 μm	4–5 per mm	11.0–13.0 × 8.0–9.5	Liu et al. 2019
*G.tsugae*	centrally to laterally stipitate, sub-dimidiate to dimidiate	whitish and soft corky context	60–75 × 7–10 μm	4–6 per mm	13.0–15.0 × 7.5–8.5	Murrill 1902
*G.thailandicum*	stipitate, greyish-red to brownish-red	context mostly brownish-red to reddish-brown	clavate to narrowly clavate, tuberculate	4–8 per mm	6.8–10.2 × 5.8–7.7	Luangharn et al. 2019

Morphologically, *G.esculentum* resemble *G.kunmingense* by radially rugose, the pileus and slender stipe (Table 2). However, *G.kunmingense* has narrower hyphae, tissues not darkening in KOH, and broadly ellipsoid to sub-globose basidiospores (7.5–10.5 × 6.0–9.0 µm, Zhao et al. 1989). In addition, *G.esculentum* shares also similarities with *G.neojaponicum* but the latter has a double-layered context with the paler layer near the pileus surface and wider basidiospores than those of *G.esculentum* (9.1–13.5 × 5.7–8.9 µm, Imazeki et al. 1939; Hapuarachchi et al. 2019).

## Supplementary Material

XML Treatment for Ganoderma dianzhongense

XML Treatment for Ganoderma esculentum

## Supplementary materials

Supplementary material 1 Phylogenetic sequence dataset Authors: Jun He Data type phylogenetic data Explanation note Sequence data of three partial loci internal transcribed spaces region (ITS), RNA polymerase II subunit 2 (RPB2), and translation elongation factor 1-alpha (TEF1-α) were used in the phylogenetic analyses. https://binary.pensoft.net/file/610288This dataset is made available under the Open Database License (http://opendatacommons.org/licenses/odbl/1.0/). The Open Database License (ODbL) is a license agreement intended to allow users to freely share, modify, and use this Dataset while maintaining this same freedom for others, provided that the original source and author(s) are credited.
